# Supplementary material for: Circadian clock regulates hepatic polyploidy by modulating Mkp1-Erk1/2 signaling pathway
Source: Nat Commun. 2017 Dec 21;8:2238. doi: 10.1038/s41467-017-02207-7 (PMC5740157; doi:10.1038/s41467-017-02207-7)
Supplement: Supplementary file 1 — Supplementary Information [file 41467_2017_2207_MOESM1_ESM.pdf]

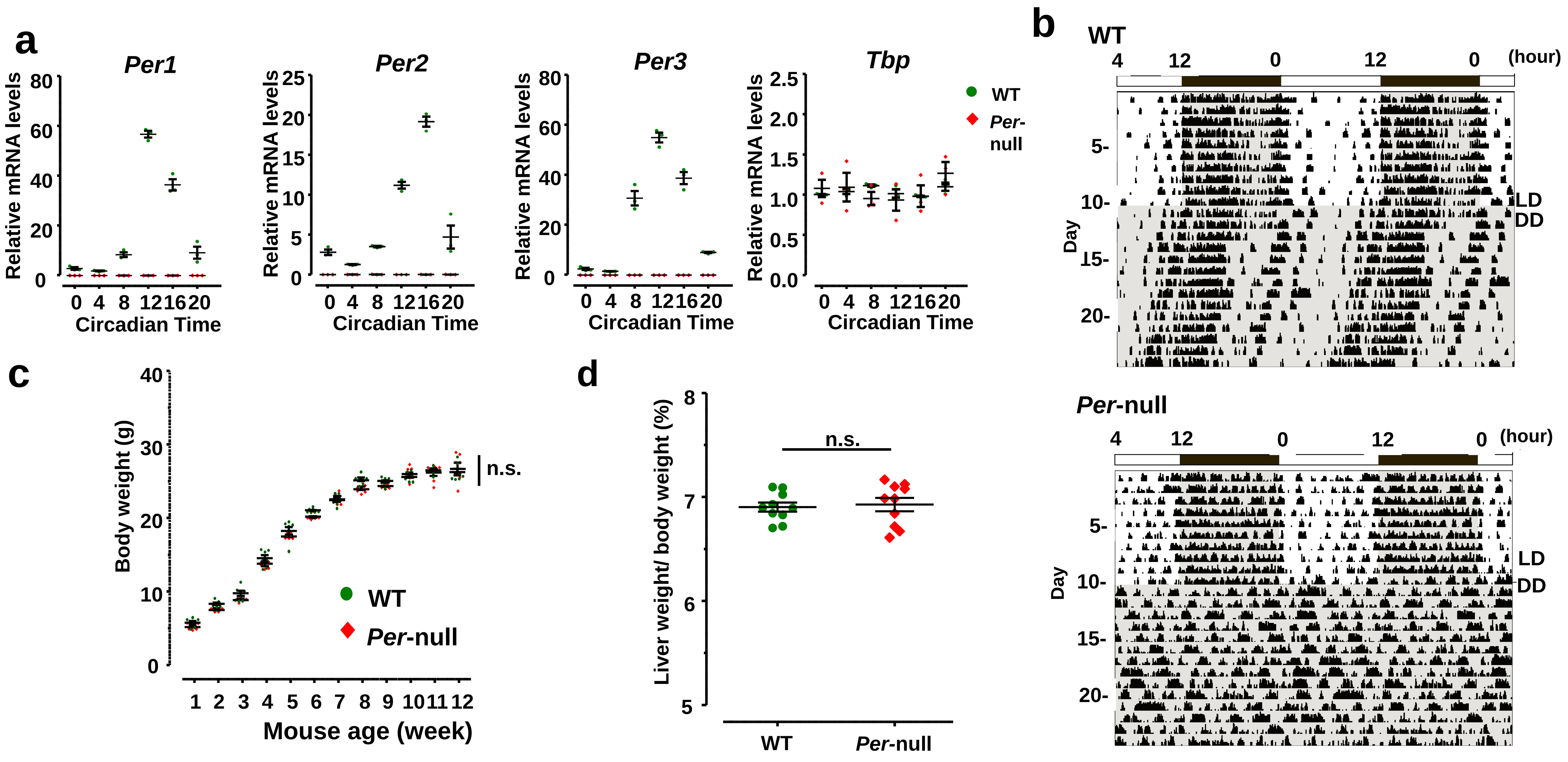

Supplementary Figure 1. Characterization of the basic physiological and anatomical profiles of *Per*-null mice.
(**a**) qRT-PCR analysis showing the expression of *Per1*, *Per2* and *Per3* mRNA in WT and *Per*-null liver at indicated times. Note clear circadian rhythms of *Period* genes in WT are completely abolished in *Per*-null mice. Values represent the mean ± SEM. (n = 3). (**b**) Representative double-plotted actogram of WT and *Per*-null mice. Mice (n = 10 at each genotype) were initially housed in a 12L:12D light-dark cycle (LD; light on 8:00 and light off at 20:00) and subsequently transferred to constant darkness (DD). WT mice entrained their activity rhythms to the environmental LD cycles, and showed a clear circadian rhythmicity (period length: 23.72 ± 0.23 h, n = 10) in DD conditions. Note that *Per*-null mice, which lack the clock oscillatory machinery, showed no rhythms in DD conditions but still displayed day-night difference of activity in LD cycle due to the masking effect of light. White and gray background indicates lights on and off, respectively. (**c**) The change of body weight of WT and *Per*-null mice during postnatal development. *Per*-null mice did not show any prominent abnormality in development, and developmental weight gain was virtually identical to that of wild-type mice, when fed a normal diet. Mean ± SEM. (n = 7); n.s., statistically not significant. (**d**) The profile of body-weight- normalized liver weight of WT and *Per*-null mice maintained on a standard diet sampled at 12 weeks of age. Mean ± SEM.; Student's *t*-test (n = 10). n.s., statistically not significant. *Tbp*, TATA-box binding protein.

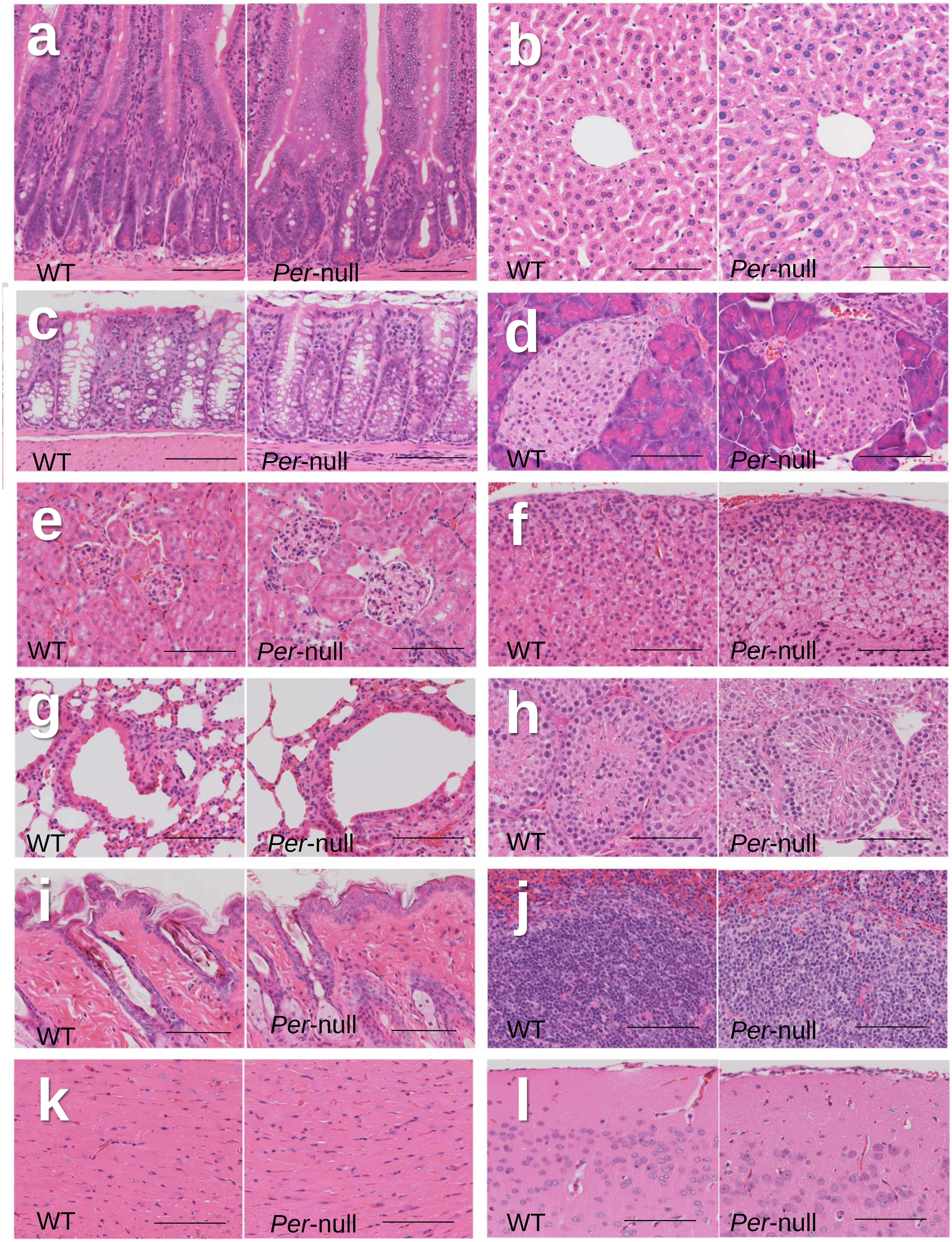

Supplementary Figure 2. Histological sections in various organs in WT and *Per*-null mice.
We performed hematoxylin and eosin staining in (**a**) small intestine, (**b**) liver, (**c**) large intestine (colon), (**d**) pancreas (exocrine gland and islet of Langerhans), (**e**) kidney (cortex), (**f**) adrenal (cortex, (**g**) lung (alveolus and bronchial epithelium), (**h**) testis, (**i**) skin (epithelium and hair follicle), (**j**) spleen (white pulp and red pulp), (**k**) cardiac muscle, and (**l**) cerebral cortex in WT and *Per*-null mice. Paraformaldehyde-fixed paraffin embedded sections (5 µm in thickness) were histologically analyzed by light-microscopy (Olympus). Note no polyploidy was observed in all tissue examined except liver. Scale bars, 100 µm.

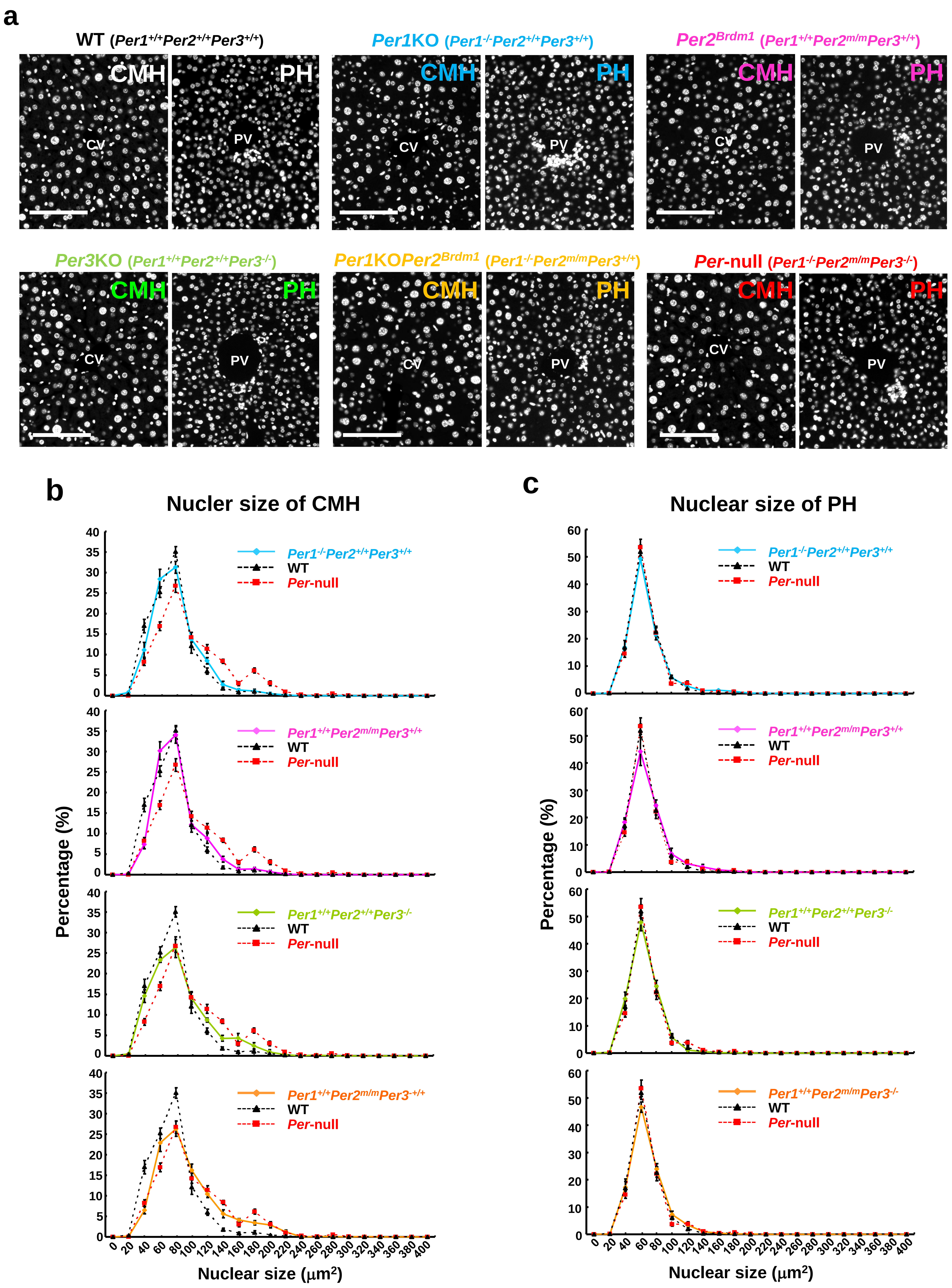

Supplementary Figure 3. Effect of *Period* genotype on nuclear size of hepatocytes *in vivo*.
(**a**) Representative examples of Hoechst-33342 stained nuclei around central vein (CV) and portal vein (PV) in livers from WT and indicated *Period* mutant mice. Quantitative data show the frequency distribution of nuclear size of centro-mid lobular hepatocytes (CMH) (**b**) and periportal hepatocytes (PH) (**c**) analyzed by Hoechst-33342 histological staining. Note genotype specific enlargement occurs only in CMH but not in PH. Values are mean ± SEM., n > 1500 cells, 3 experiments. Scale bars, 100 μm.

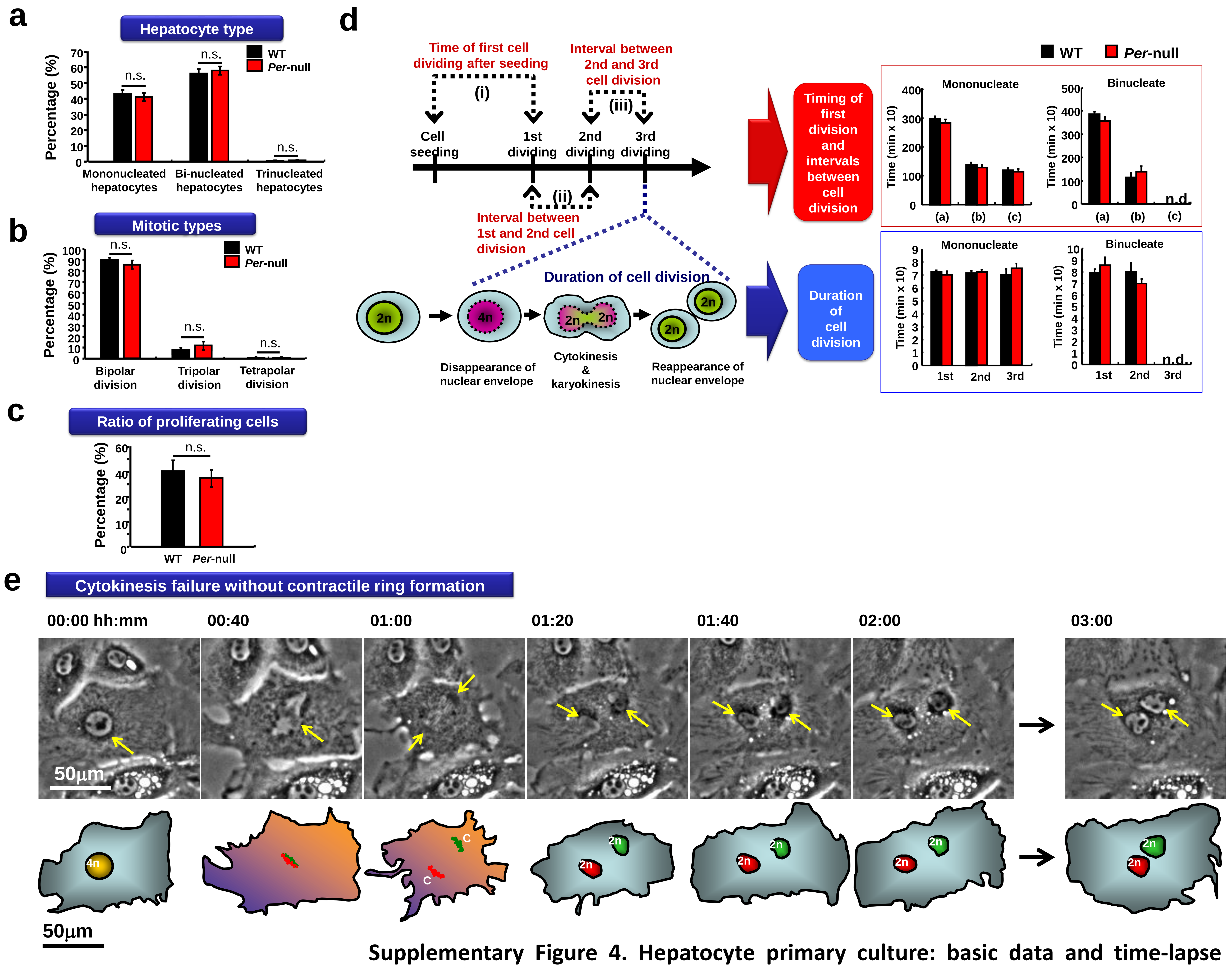

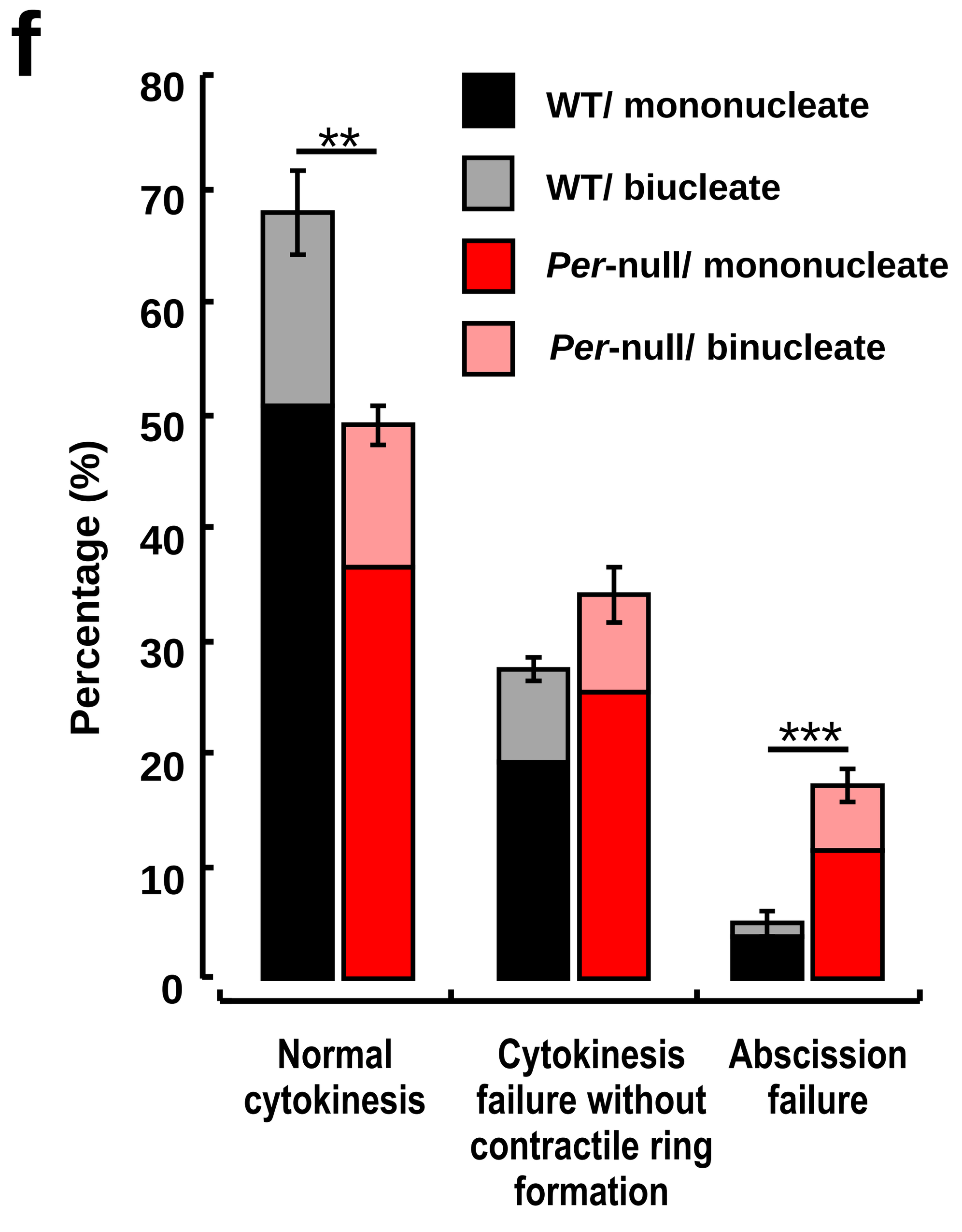

Supplementary Figure 4. Hepatocyte primary culture: basic data and time-lapse images of hepatocyte primary culture.

(**a-d**) Quantitative analyses of cell types (**a**), division types (**b**), and the ratio of proliferating cells (**c**) from time-lapse images of hepatocytes in culture. The percentage of mono-, bi-, and tri- nucleate hepatocytes (**a**), the percentage of bipolar, tripolar and tetrapolar division of hepatocytes (**b**), and the percentage of proliferating cells (**c**) show no difference between WT and *Per*-null hepatocytes. (**d**) Detailed analysis of mitotic events in hepatocytes in primary culture. Schematic presentation of time course of cultured hepatocytes (left-upper figure) and the third mitotic events (left-lower figure). After cell seeding, mitotic events were observed for 3 times, and we measured intervals between these events. (i) indicates the time of first cell division after seeding, (ii) indicates the interval between first and second cell division, and (iii) indicates the interval between second and third cell division. We also measured the duration of cell division, which indicates the time between the disappearance and reappearance of the nuclear envelope in the first, the second and the third mitosis. Above quantification was performed in each cell type (mono- and bi-nucleate hepatocytes). Note the interval time between each cell division (upper panel), and duration of cell division (lower panel) have no significant difference between WT and *Per*-null hepatocytes. All data in (**a-d**) strongly suggest that there are no genotype-specific basic mitotic events in the present culture system. Values are mean ± SEM. (WT, n = 526; *Per*-null, n = 717; 5 experiments). n.s., statistically not significant. n.d., not detected. (**e**), Representative time-lapse images of cultured hepatocytes showing a cytokinesis failure without contractile ring formation. We classified cytokinesis of hepatocytes into three groups: (1) normal cytokinesis, (2) cytokinesis failure without contractile ring formation, and (3) abscission failure (see **Supplementary Movie 1**). (1) and (3) are presented in **Fig. 3**, and here (2) is shown: Yellow arrows indicate the localization of nuclei and chromosomes of dividing hepatocytes. Cartoons corresponding to time-lapse images are shown with the number of chromosomes (c) in the gametes (n). (**f**) Quantification of the percentage of three types of cytokinesis from time-lapse imaging: (1) normal cytokinesis, (2) cytokinesis failure without contractile ring formation, and (3) abscission failure. Among three groups, only the abscission failure is increased in *Per*-null hepatocytes compared to WT. Values represent the mean ± SEM., $**P<0.01$, $***P<0.001$ by Student's unpaired t-test (WT, n = 526 cells; *Per*-null, n = 717 cells from 5 experiments for each group. *Continued to next page.*

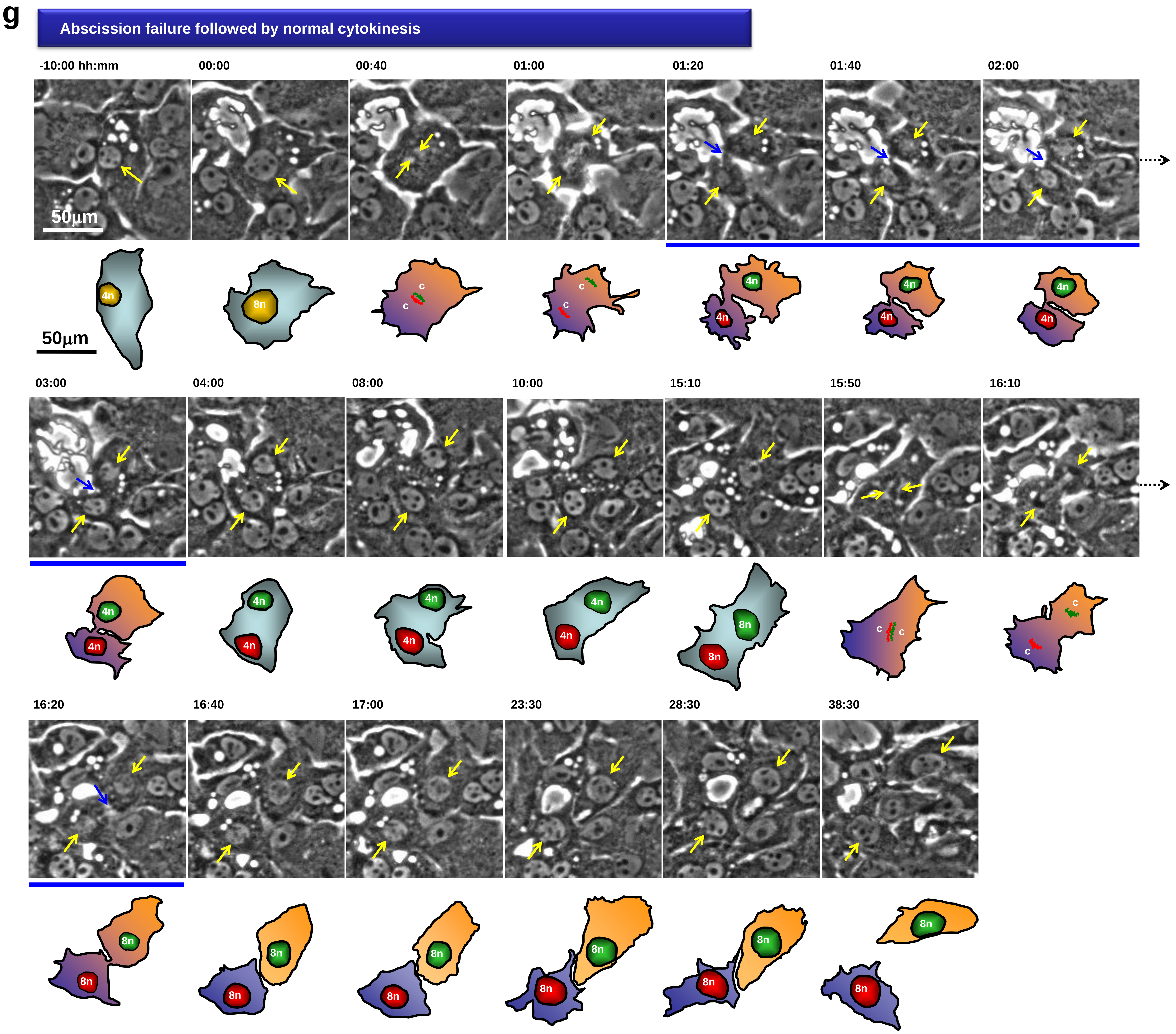

Supplementary Figure 4. (continued)

(**g**) Representative time-lapse images of cultured hepatocytes showing formation of enlarged nucleus with an abscission failure followed by a normal cytokinesis (see **Supplementary Movie 2**). First, the cell with a 4n (based on Hoechst staining) nucleus entered into S-phase to become 8n, then progressed to M-phase but failed abscission, resulting in a binuclear hepatocyte (4n × 2). This cell then started a second S-phase (8n × 2), went into a second M-phase with both nuclei fusing, performed cytokinesis successfully, and then became 2 mononucleated hepatocytes each with a single enlarged nucleus (8n × 1; 2 cells). Thus, two cell cycles, the first failing abscission, lead to polyploid hepatocytes with a single enlarged nucleus. Yellow arrows indicate the localization of nuclei and chromosomes of dividing hepatocytes. Blue arrows exhibit intercellular bridges between daughter cells. Below, cartoons corresponding to time-lapse images are shown, demarcating copy number of chromosome. The states of intercellular bridge (around 20-40 min) between daughter cells are blue-underlined. See text for further details. Scale bars (**e, g**), 50 µm.

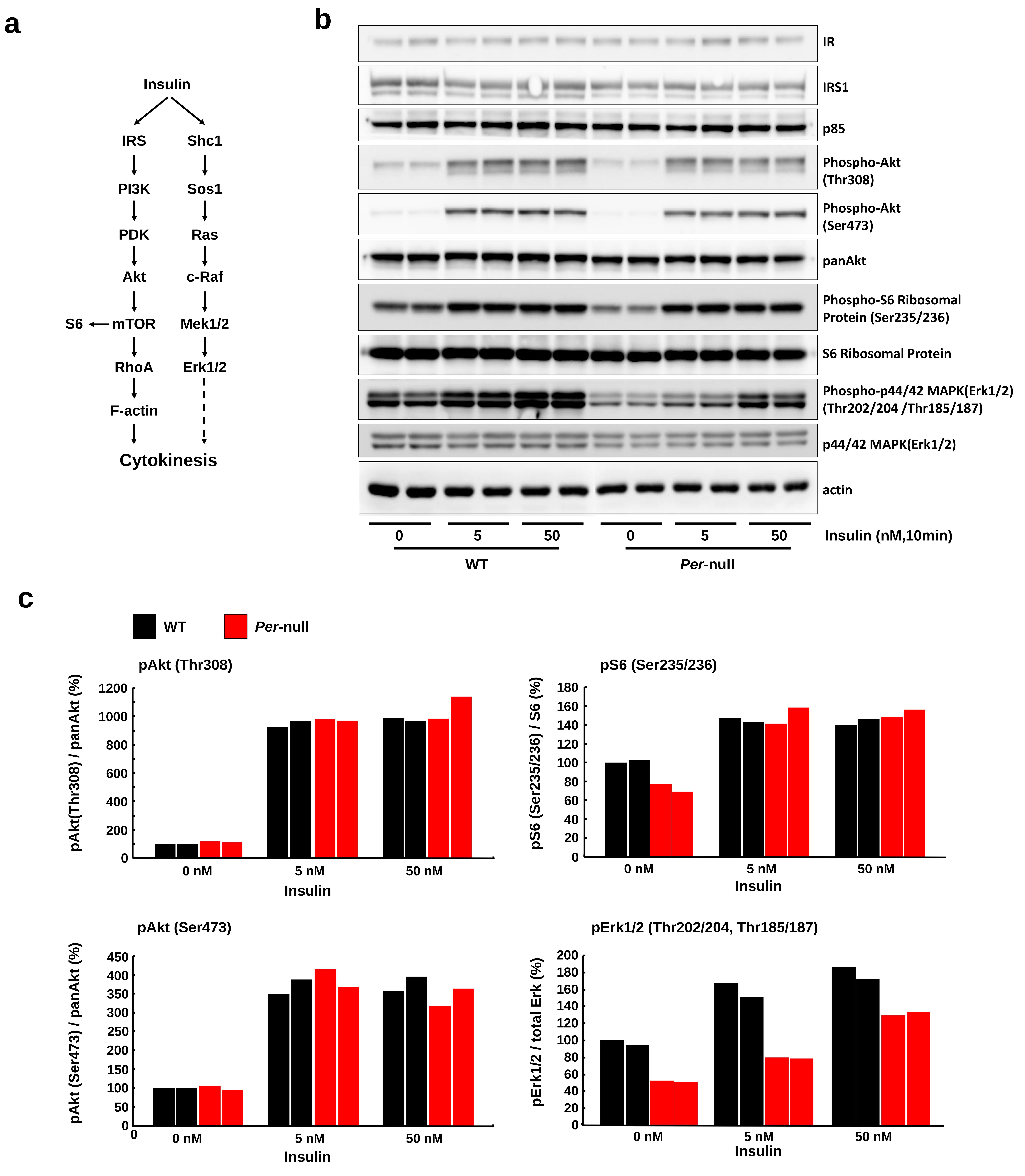

Supplementary Figure 5. Kinase activities in insulin signaling pathways in cultured hepatocytes: genotype specificity.
(**a**) Schematic representation showing two major insulin downstream signaling pathways in liver. (**b**) Immunoblots of cultured hepatocytes with insulin treatment as indicated. (**c**) Quantitative data displayed the level of phosphorylation of Akt (Thr308 and Ser473), S6 (Ser235/236), and Erk1/2 (Thr202/204, Thr185/187). Although insulin dose-dependently activates all these kinases in both genotypes, the extent of activation of pErk1/2 was severely damped in *Per*-null hepatocytes. Duplicate experiments showing indicated value of one sample at 0 nM insulin treatment being 100 %.

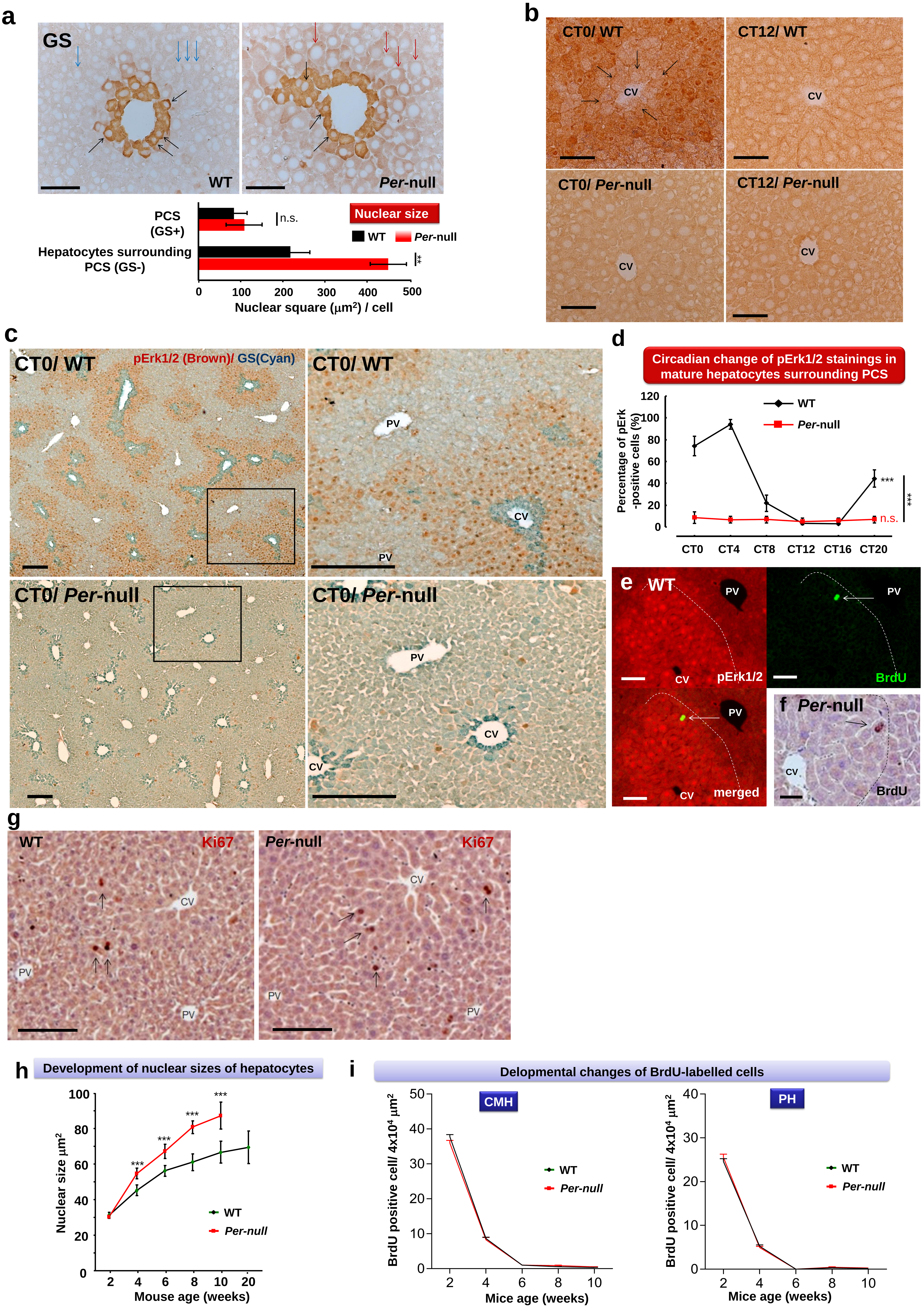
a
GS
WT
Per-null
PCS (GS+)
Hepatocytes surrounding PCS (GS-)
n.s.
**
Nuclear size
WT
Per-null
0 100 200 300 400 500
Nuclear square (μm²) / cell
b
CT0/ WT
CT12/ WT
CT0/ Per-null
CT12/ Per-null
CV
c
CT0/ WT
pErk1/2 (Brown)/ GS(Cyan)
CT0/ Per-null
PV
CV
d
Circadian change of pErk1/2 stainings in mature hepatocytes surrounding PCS
Percentage of pErk -positive cells (%)
WT
Per-null
***
n.s.
CT0 CT4 CT8 CT12 CT16 CT20
e
WT
PV
CV
pErk1/2
BrdU
merged
f
Per-null
BrdU
g
WT
Per-null
Ki67
h
Development of nuclear sizes of hepatocytes
Nuclear size μm²
Mouse age (weeks)
WT
Per-null
***
i
Delopmental changes of BrdU-labelled cells
CMH
PH
BrdU positive cell/ 4x10⁴ μm²
Mice age (weeks)
WT
Per-null

Supplementary Figure 6. Distribution of pErk1/2 in pericentral stem cells (PCS) and its neighboring mature hepatocytes.

(**a**) Pericentral stem cells (PCS) are marked by glutamine synthetase (GS) (Wang et al. 2015). PCS labeled by GS were distributed around CV in both WT and *Per*-null mice. Note the nuclear size of PCS were two-third diploid and one-third tetraploid (4n) in WT (black arrows, left figure), and this tendency does not change in *Per*-null hepatocytes (black arrows, right figure). In contrast, hepatocytes surrounding PCS, presumably the descendants of PCS (Wang et al. 2015), showed mostly 4n in WT (blue arrows, left figure), but mostly 8n-16n high polyploidy in *Per*-null hepatocytes (red arrows, right figure). Lower graph represents the quantification of nuclear sizes in PCS and hepatocytes surrounding PCS. Note the nuclear sizes of PCS do not change between two genotypes, although there is marked enlargement of mature hepatocytes in *Per*-null strain. (**b**) pErk1/2 immunohistochemistry near CV region at CT0 and CT12 in WT and *Per*-null mice. Note the PCS cells show faint expression of pErk1/2 even at CT0, the peak time of pErk1/2 expression. (**c**) Double staining of pErk1/2 (brown) and GS (cyan), a marker of hepatocytes surrounding the central vein (CV), in liver of WT and *Per*-null mice. Note that pErk1/2 was expressed in centro-midlobular hepatocytes (CMH) centering the CV, but not in periportal hepatocytes (PH) surrounding the portal vessels (PV) in WT mice at CT0. However, pErk1/2 signals in CMH hepatocytes were no longer observed in *Per*-null mice, although glutamine synthetase was not altered in either genotype. (**d**) Quantitative immunohistochemical analysis of circadian expression of pErk1/2 positive cells in mature hepatocytes surrounding PCS. Percentage of pErk1/2 positive cells in mature hepatocytes surrounding PCS were counted in WT (black) and *Per*-null mice (red) at 4 h intervals in constant dark conditions. Values represent the mean ± SEM., ****P*<0.001 (n = 3 for each time point in WT and *Per*-null mice). n.s., no statistical significance. (**e**) Dual immunofluorescence with BrdU (green) and pErk1/2 (red) antibodies in WT liver. Their merged image is shown in lower left. Arrows indicate that a pErk1/2 cell is in S-phase. BrdU (0.15 mg/g body weight; Sigma, St. Louis, MO) was injected at 10 weeks of age 1hr before sacrifice at dawn (ZT0). Note BrdU labeled cell was on the peripheral border (CV side) of pErk1/2 expressing cell group in hepatic lobule. (**f**) Location of BrdU positive cells in *Per*-null liver. For comparison, we also checked BrdU labelling (DAB; arrow) in *Per*-null hepatocytes, but, since *Per*-null hepatocytes do not show pErk1/2 immunoreactivity, it is impossible to reliably relate the two. In single stained histological sections, however, we found that BrdU cells were also located on the peripheral border of large sized polyploid cells. (**g**) Ki67-immunopositive cells in the hepatic lobule (**h**) of WT and *Per*-null adult mice. (**h, i**) Quantitative analyses of the development of nuclear sizes (**h**) and the number of BrdU-incorporated cells (**i**) in WT and *Per*-null liver (mean ± SEM; n=5 at each developmental stage for each genotype; injection protocol and tissue preparation are the same as (**e**). (**i**) In both genotypes, the average nuclear size increases according to the development from 2 to 10 weeks. Although there was no significant difference between genotype in 2 weeks old mice (see also **Fig. 1g**), the increase of nuclear ploidy became prominent in 4 weeks-old *Per*-null mice, and further increased until 10 weeks. In two-way ANOVA, the main effect of time and genotype were both significant. (**j**) Developmental analysis demonstrated that the sharp age-dependent decline of BrdU labelling in both genotypes: the actual labelling of hepatocytes decrease more than 50 times by 10 weeks, compared to 2 weeks. In two-way ANOVA, only the main effect of time was significant in CMH and PH. These data clearly demonstrated that age-dependent increase in cell size (showing the degree and the proportion of polyploidy cells) is inversely proportional to the number of proliferating cells. Scale bar, Bars = 20 μm (**e**), 50 μm (**a, b, f**), 200 μm (**c, g**). See also the following **Supplementary Discussion for Fig. 6**.

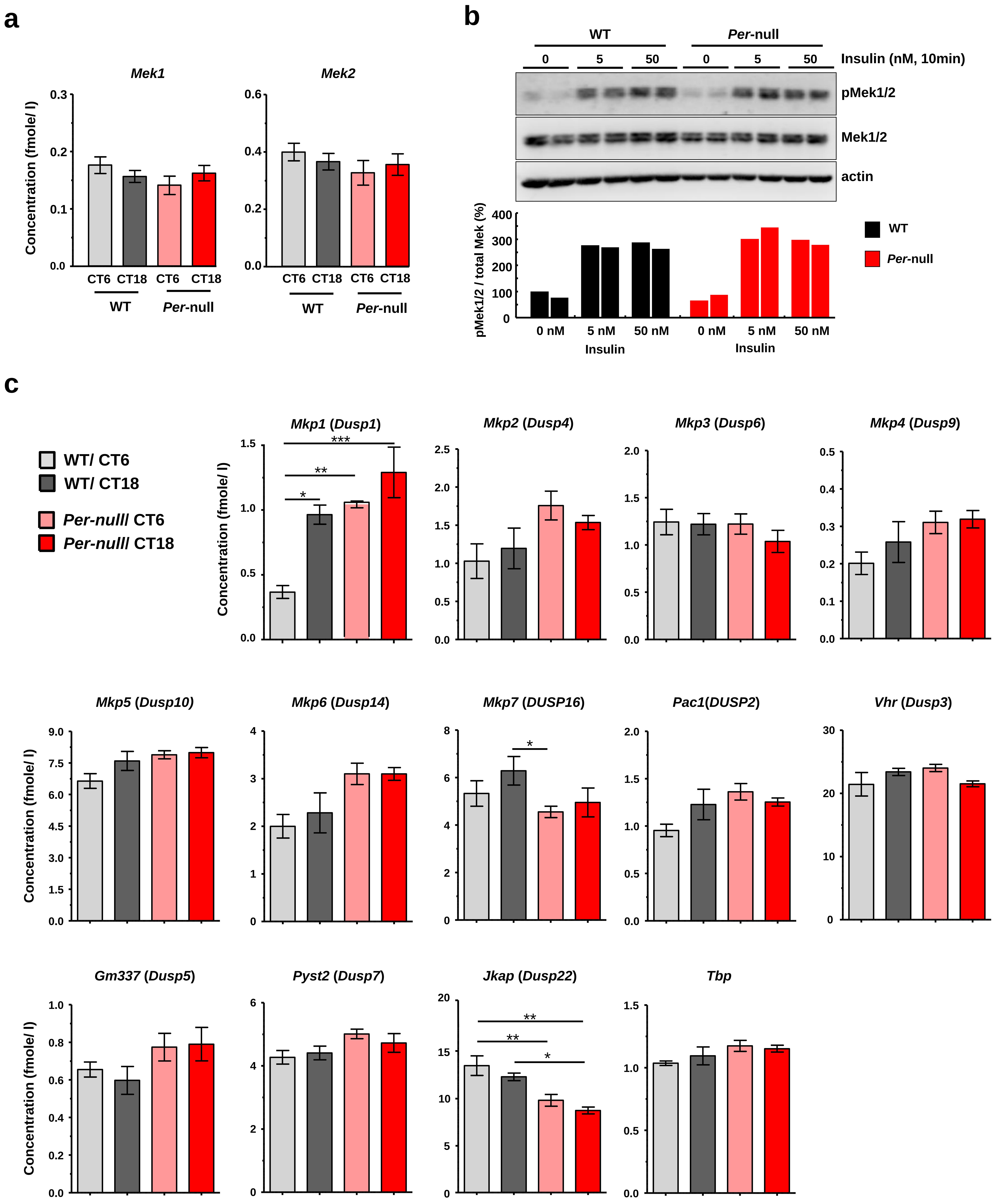

Supplementary Figure 7. Enzymes regulating Erk1/2 phosphorylation in hepatocytes.
(**a**) qPCR analyses of Erk1/2 up-stream kinases Mek1 and Mek2 mRNA at subjective day (CT6) and subjective night (CT18) in WT and *Per*-null livers. Neither Mek1 nor Mek2 showed significant difference for day-night and genotypes. (**b**) Immunoblots of Mek1/2 kinases in cultured hepatocytes after treatment with insulin. Although Mek1/2 phosphorylation is sensitive to insulin treatment, there was no genotype difference at each concentration of insulin. (**c**) qPCR analyses of Erk phosphatases, namely Mkp family, at CT6 and CT18 in WT and *Per*-null livers. Note that *Mkp1* was the only gene showing daily rhythm in WT, and increased in *Per*-null mice. Values are the mean ± SEM. (n = 4), *$P < 0.05$, **$P < 0.01$, ***$P < 0.001$.

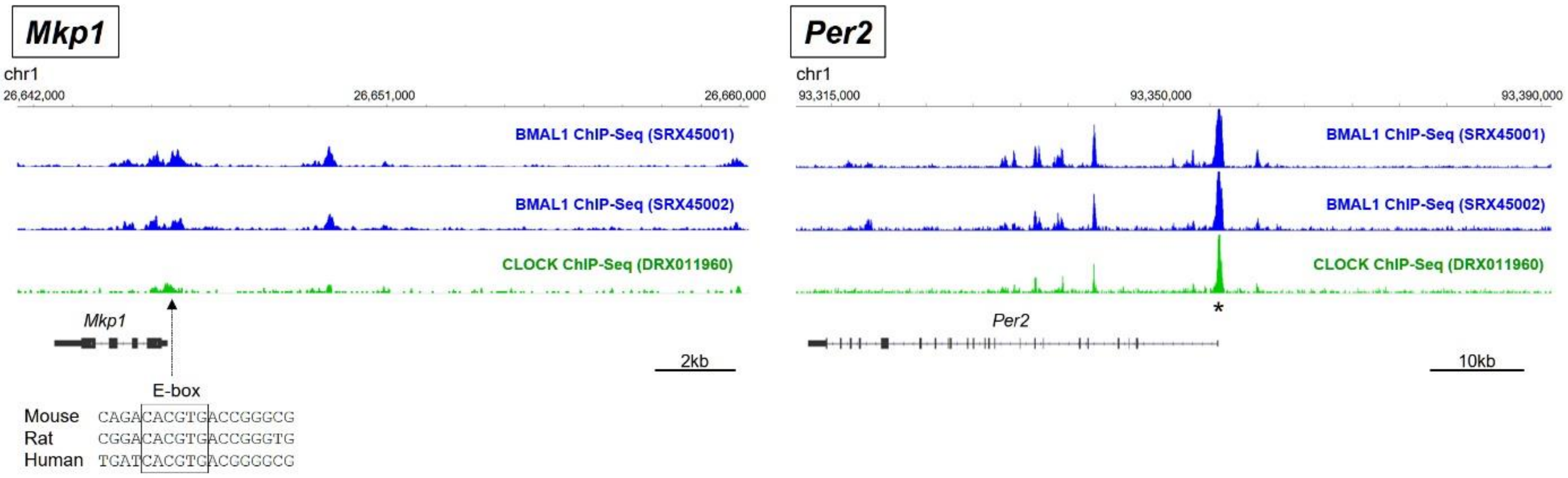

Supplemental Figure 8. Visualization of previously reported ChIP-seq signals for BMAL1 (blue) and CLOCK (green) on Mkp1 (Dusp1) and Per2.

Here we present the previously reported Mkp1 (Dusp1) and Per2 data on ChIP-seq signals for BMAL1 (blue) by Annayev et al. in J Biol Chem 289, 5013-24, 2014 (SRA ID: SRX45001 and SRX45002); and CLOCK (green) by Yoshitane et al. in Mol Cell Biol 34, 1776-87, 2014 (SRA ID: DRX011960). In both reports, livers at ZT8 were analyzed, and peak calling analysis revealed significant peaks in the proximity of this E-box.

BigWig files that correspond to each ChIP-seq experiment were downloaded from the ChIP-Atlas website (http://chip-atlas.org/) and visualized with the Integrative Genomics Viewer (http://software.broadinstitute.org/software/igv/). Arrow indicates the position of the Mkp1 E-box, to which the binding of CLOCK and BMAL1 has been confirmed in ChIP-seq experiments, albeit with lower affinity than to the *Per2* E'-box (asterisk).

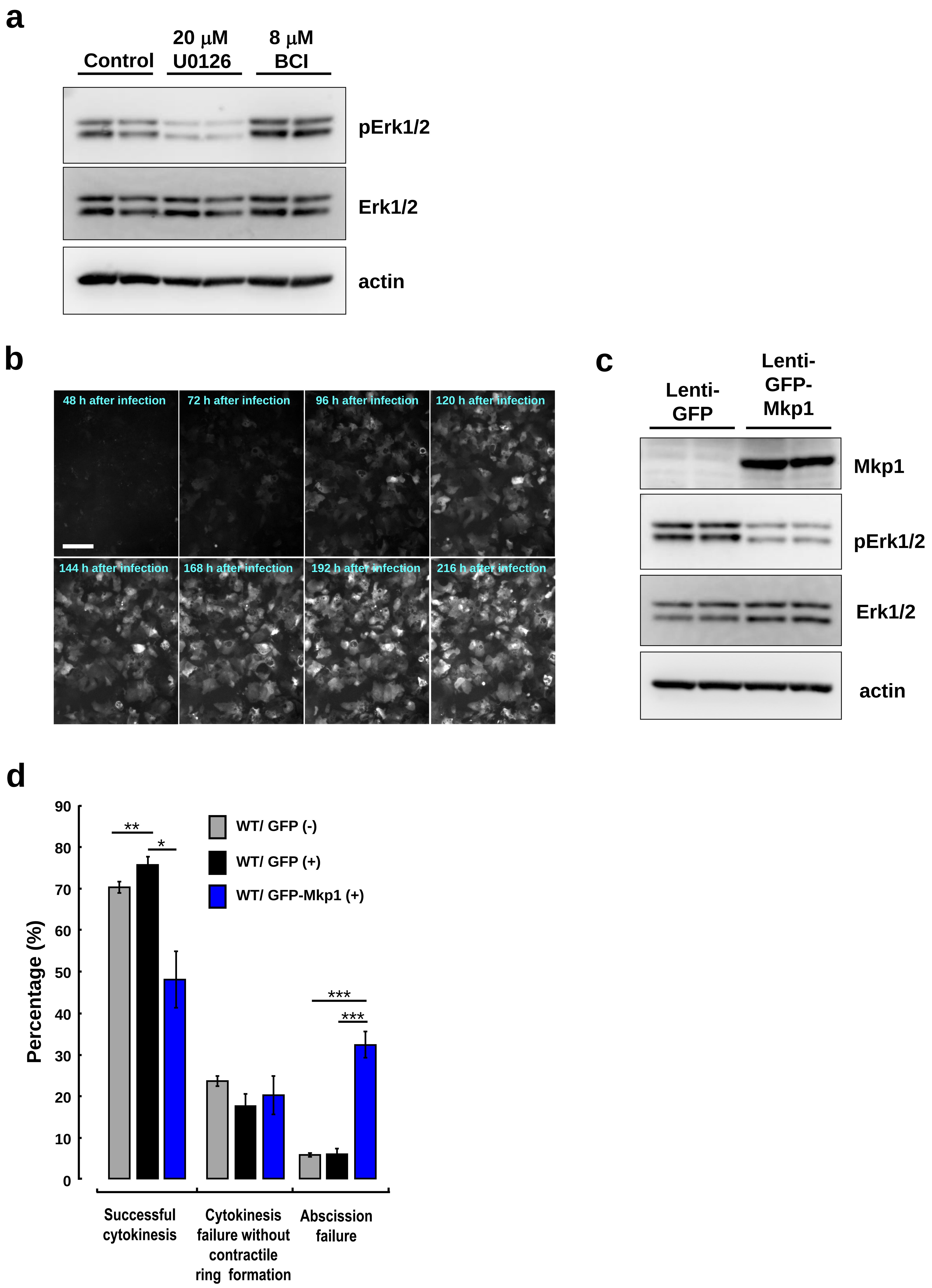

Supplementary Figure 9. Regulation of Erk1/2 phosphorylation in cultured hepatocytes: pharmacological regulation and lentivirus-mediated Mkp1 transduction.
(**a**) pErk1/2 and total Erk1/2 after treatment with MEK inhibitor (U0126) or MKP1 inhibitor (BCI) in hepatocytes isolated from WT liver. Note that pErk1/2 decreases after U0126 treatment, and increases after BCI treatment. (**b, c**) pErk1/2 and total Erk1/2 after the overexpression of GFP-labeled Mkp1 to WT hepatocytes by lentiviral vector. Time-course images of GFP expression after the transduction with lentivirus carrying GFP-labeled Mkp1 (Lenti-GFP-Mkp1) (**b**) Immunoblots of Mkp1 from cultured hepatocytes transduced with Lenti-GFP-Mkp1 or with control lentivirus carrying GFP (Lenti-GFP). (**c**) Protein lysates were extracted from the hepatocytes after 192 h of virus infection. (**d**) Quantification of three types of cytokinesis after the overexpression of Lenti-GFP-Mkp1 or Lenti-GFP in cultured hepatocytes (WT/GFP(-), n = 257, WT/GFP(+) n = 209, WT/GFP-Mkp1(+), n = 189; 3 experiments for each group). All values represent the mean ± SEM. **P*<0.05, ***P*<0.01, ****P*<0.001. Scale bar, 400 μm.

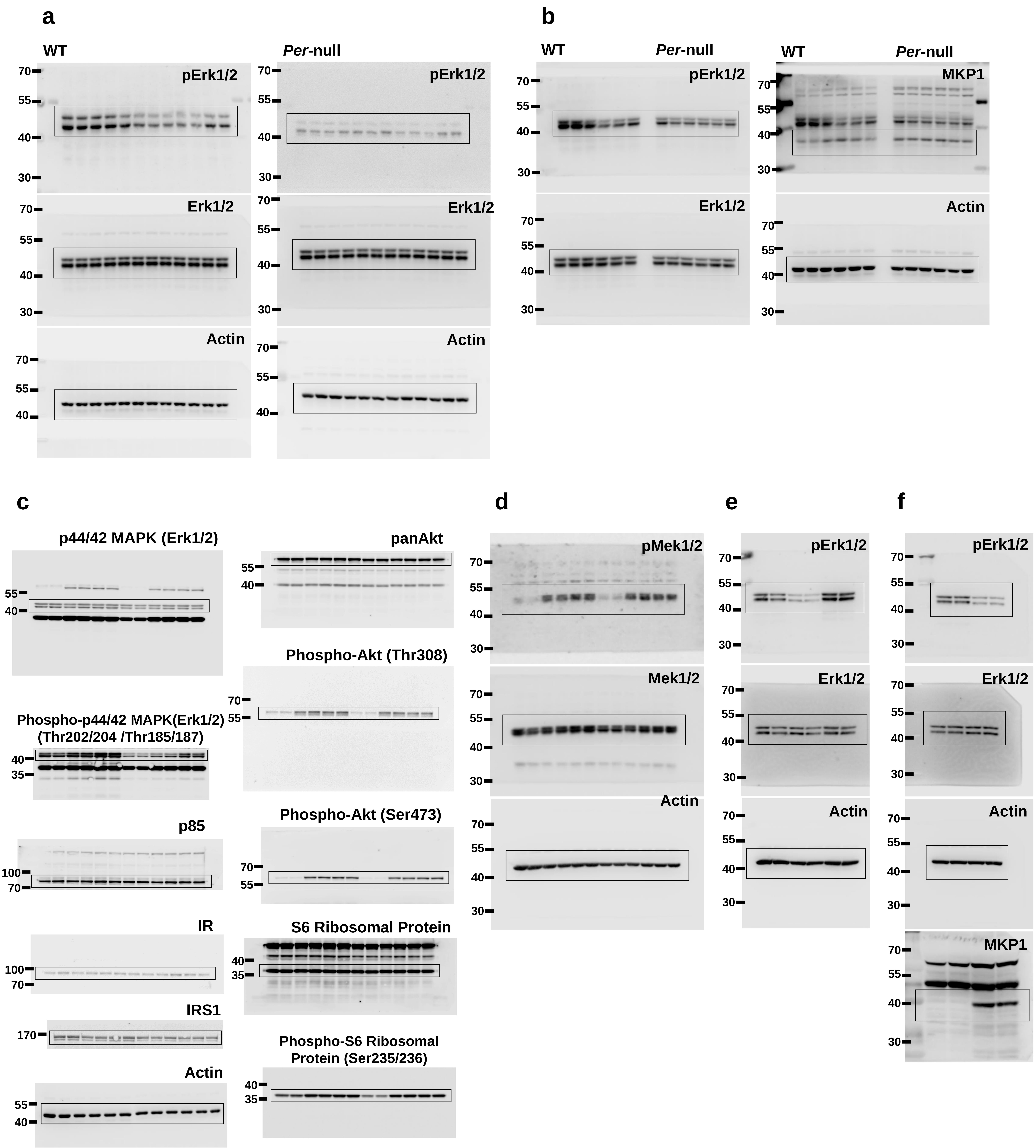

Supplementary Figure 10.
Images of uncropped Western blots shown in Fig. 4a (a), Fig. 5b (b), Supplementary Fig. 5b (c), Supplementary Fig. 7b (d), Supplementary Fig. 9a (e), and Supplementary Fig. 9b (f).

Supplementary Discussion

Difference of pErk1/2 expression in pluripotent pericentral stem cells (PCS) and mature hepatocytes surrounding PCS. It is interesting to note that the Mkp1-pErk1/2 pathway appears weak in pericentral stem cells (PCS) (Wang et al. 2015) lining the central vein, but more active in mature CMH surrounding PCS, which are possible descendants of PCS (Wang et al. 2015). Since Ki67-expressing cells, representing the cells in growth fraction (cells out of G_0), were sparsely distributed but dominantly located in the midlobular region **(Supplementary Fig. 6g),** the mature CMH thus appear to play a central role in homeostatic renewing process of organ mass, in contrast to pluripotent PCS stem cells. The separation of pluripotent stem cells and proliferative cells is commonly known in most tissues such as small intestine[14]. Here, proliferative hepatocytes were dispersed among silent G_0 hepatocytes **(Supplementary Fig. 6g)**. Although a correlation between rate of proliferation and liver polyploidization has been reported[25, 27, 71], no such correlation was found in *Per*-null liver (**Supplementary Fig. 6i**).

Supplementary References

71 Gupta, S. Hepatic polyploidy and liver growth control. *Semin Cancer Biol.* **10**, 161-171 (2000)
