# Supplementary material for: Circadian clock regulates hepatic polyploidy by modulating Mkp1-Erk1/2 signaling pathway
Source: Nat Commun. 2017 Dec 21;8:2238. doi: 10.1038/s41467-017-02207-7 (PMC5740157; doi:10.1038/s41467-017-02207-7)
Supplement: Supplementary file 3 — Description of Additional Supplementary Files [file 41467_2017_2207_MOESM3_ESM.pdf]

Description of Additional Supplementary Files

File Name: Supplementary Movie 1
Description:

This video shows three types of cytokinesis: the first is normal (successful) cytokinesis, the second is cytokinesis failure without contractile ring formation (no cytokinesis), and the third is abscission failure at the last step of cytokinesis. In each type of cytokinesis, cell boundary was demarcated with red broken lines. Blue arrows indicate intercellular bridges. See also Fig. 3a, 3b and Supplementary Figure 4e.

File Name: Supplementary Movie 2
Description:

Formation of polyploid cell with enlarged nucleus: abscission failure followed by successful cytokinesis. See also Supplementary Figure 4g.

File Name: Supplementary Movie 3
Description:

Mitosis of WT hepatocytes after lentivirus-mediated overexpression. Lentiviral infected cells are marked green. The first shows cells transduced with GFP showing successful cytokinesis, and the second shows cells transduced with GFP-Mkp1 showing abscission failure. See also Fig. 5e.
